# Assessing the Performance of Hierarchical Forecasting Methods on the Retail Sector

**DOI:** 10.3390/e21040436

**Published:** 2019-04-24

**Authors:** José Manuel Oliveira, Patrícia Ramos

**Affiliations:** 1 INESC Technology and Science, Rua Dr. Roberto Frias, 4200-465 Porto, Portugal; 2 Faculty of Economics, University of Porto, Rua Dr. Roberto Frias, 4200-464 Porto, Portugal; 3 School of Accounting and Administration of Porto, Polytechnic Institute of Porto, Rua Jaime Lopes Amorim, 4465-004 S. Mamede de Infesta, Portugal

**Keywords:** hierarchical forecasting, information criteria, entropy, model selection, ARIMA, state space models, retail

## Abstract

Retailers need demand forecasts at different levels of aggregation in order to support a variety of decisions along the supply chain. To ensure aligned decision-making across the hierarchy, it is essential that forecasts at the most disaggregated level add up to forecasts at the aggregate levels above. It is not clear if these aggregate forecasts should be generated independently or by using an hierarchical forecasting method that ensures coherent decision-making at the different levels but does not guarantee, at least, the same accuracy. To give guidelines on this issue, our empirical study investigates the relative performance of independent and reconciled forecasting approaches, using real data from a Portuguese retailer. We consider two alternative forecasting model families for generating the base forecasts; namely, state space models and ARIMA. Appropriate models from both families are chosen for each time-series by minimising the bias-corrected Akaike information criteria. The results show significant improvements in forecast accuracy, providing valuable information to support management decisions. It is clear that reconciled forecasts using the Minimum Trace Shrinkage estimator (MinT-Shrink) generally improve on the accuracy of the ARIMA base forecasts for all levels and for the complete hierarchy, across all forecast horizons. The accuracy gains generally increase with the horizon, varying between 1.7% and 3.7% for the complete hierarchy. It is also evident that the gains in forecast accuracy are more substantial at the higher levels of aggregation, which means that the information about the individual dynamics of the series, which was lost due to aggregation, is brought back again from the lower levels of aggregation to the higher levels by the reconciliation process, substantially improving the forecast accuracy over the base forecasts.

## 1. Introduction

Retailers need demand forecasts at different levels of aggregation to support decision-making at operational and short-term strategic levels [1]. Consider a retailer warehouse storing inventory that is used to replenish multiple retail stores: Store-level forecasts at different product levels are needed to manage inventory in the store or to allocate shelf space, but aggregate forecasts are also required for the inventory decisions of the retailer warehouse [2]. Understanding whether these aggregate forecasts should be generated independently at each level of the hierarchy, based on the aggregated demand, or obtained using an hierarchical forecasting method, which depends on the aggregation constraints of the hierarchy but ensures coherent decision-making at the different levels, is the gap we seek to address in this paper.

SKUs (Stock Keeping Units) are naturally grouped together in hierarchies, with the individual sales of each product at the bottom level of the hierarchy, sales for groups of related products (such as categories, families, or areas) at increasing aggregation levels, and the total sales at the top level [3]. Generating accurate forecasts for hierarchical time-series can be particularly difficult. Time-series at different levels of the hierarchical structure have different scales and can exhibit very different patterns. The time-series at the most disaggregated level can be very noisy and are often intermittent, being more challenging to model and forecast. Aggregated series at higher levels are usually much smoother and, therefore, easier to forecast. Additionally, in order to ensure coherent decision-making at the different levels of the hierarchy, it is essential that forecasts of each aggregated series be equal to the sum of the forecasts of the corresponding disaggregated series. However, it is very unlikely that these aggregation constraints will be satisfied if the forecasts for each series in the hierarchical structure are generated independently. Finally, hierarchical forecasting methods should take advantage of the interrelations between the series at each level of the hierarchy.

The most traditional approaches to hierarchical forecasting are bottom-up and top-down methods. The bottom-up method involves forecasting each series at the bottom level, and then summing these to obtain forecasts at the higher levels of the hierarchy [4,5,6,7]. The main advantage of this approach is that, since forecasts are obtained at the bottom level, no information is lost due to aggregation. However, it ignores the inter-relations between the series and usually performs poorly on highly aggregated data. The top-down method involves forecasting the most aggregated series at the top level, and then disaggregating these, using either historical [8] or forecasted proportions [9], to obtain bottom level forecasts. Top-down approaches based on historical proportions tend to produce less accurate forecasts at lower levels of the hierarchy. The middle-out approach combines both bottom-up and top-down methods. First, forecasts for each series of an intermediate level of the hierarchy chosen previously are obtained. The forecasts for the series above the intermediate level are produced using the bottom-up approach, while the forecasts for the series below the intermediate level are produced using the top-down approach. Empirical studies comparing the performance of bottom-up and top-down methods have mixed results as to a preference for either bottom-up or top-down [4,6,10,11,12].

Recent work in the area tackles the problem using a two-stage approach: In the first step, forecasts for all series at all the levels of the hierarchy, rather then at a single level, are independently produced (these are called base forecasts). Then, a regression model is used to combine these to give coherent forecasts (these are called reconciled forecasts). Athanasopoulos et al. [9] and Hyndman et al. [13] used the Ordinary Least Squares (OLS) estimator and showed that their approach worked well, compared to most traditional methods. Hyndman et al. [14] suggested the Weighted Least Squares (WLS) estimator, proposing the variances of the base forecast errors as a proxy to the diagonal of the errors covariance matrix, with null off-diagonal elements. They also introduced several algorithms to make the computations involved more efficient under a very large number of series. To extend the work of Hyndman et al. [14], Wickramasuriya et al. [15] proposed a closed-form solution, based on the Generalised Least Squares (GLS) estimator, that minimised the sum of the variances of the reconciled forecast errors incorporating information from a full covariance matrix of the base forecast errors. The authors evaluated the performance of their method, compared to the most commonly-used methods and the results showed that it worked well with both artificial and real data.

Erven and Cugliari [16] proposed a Game-Theoretically OPtimal (GTOP) reconciliation method that selected the set of reconciled predictions, such that the total weighted quadratic loss of the reconciled predictions will never be greater than the total weighted quadratic loss of the base predictions. The authors illustrated the benefits of their approach on both simulated data and real electricity consumption data. This approach required fewer assumptions about the forecasts and forecast errors, but it did not have a closed-form solution and did not scale well for a huge set of time-series.

Mircetic et al. [17] proposed a top-down approach for hierarchical forecasting in a beverage supply chain, based on projecting the ratio of bottom and top level series into the future. Forecast projections were then used to disaggregate the base forecasts of the top level series. The disadvantage of all top-down approaches, including this one, is that they do not produce unbiased coherent forecasts [13].

The remainder of the paper is organized as follows. The next section presents a brief description of the two most widely-used approaches to time-series forecasting: State space models and ARIMA models. The procedure for using information criteria in model selection is also discussed. Section 3 describes the methods more commonly used to forecast hierarchical time-series. Section 4 presents the case study of a Portuguese retailer, explains the evaluation setup implemented and error measures used, and discusses the results obtained. Finally, Section 5 offers the concluding remarks.

## 2. Pure Forecasting Models

We consider two alternative forecasting methods for generating the base forecasts used by hierarchical forecasting approaches; namely, state space models and ARIMA models. These are briefly described in this section, giving a special focus on the use of information criteria for model selection.

### 2.1. State Space Models

Forecasts generated by exponential smoothing methods are weighted averages of past observations, where the weights decrease exponentially as the observations get older. The component form representation of these methods comprises the forecast equation and one smoothing equation for each of the components considered, which can be the level, the trend, and the seasonality. The possibilities for each of these components are: $\mathrm{Trend}=\left\{N,A,A_{d}\right\}$ and $\mathrm{Seasonality}=\left\{N,A,M\right\}$, where N, A, $A_{d}$ and M mean, respectively, none, additive, additive damped, and multiplicative. By considering all combinations of the trend and seasonal components, nine exponential smoothing methods are possible. Each method is usually labelled by a pair of letters, (T,S), specifying the type of trend and seasonal components. Denoting the time-series by $y_{t},\;t=1,2,\ldots ,n$ and the forecast of $y_{t+h}$, based on all data up to time *t* by ${\hat{y}}_{t+h|t}$, the component form of the additive Holt-Winters’ method, $\left(A,A\right)$, is

(1a)y^t+h|t=lt+hbt+st+h−m(k+1)(1b)lt=αyt−st−m+1−αlt−1+bt−1(1c)bt=β*lt−lt−1+1−β*bt−1(1d)st=γyt−lt−1−bt−1+1−γst−m

$$0\leq \alpha \leq 1,\;0\leq \beta^{*}\leq 1,\;0\leq \gamma \leq 1-\alpha ,$$

where $l_{t}$, $b_{t}$, and $s_{t}$ denote, respectively, the estimates of the series level, trend (slope), and seasonality at time *t*; *m* denotes the period of seasonality; and *k* is the integer part of (h−1)/m. The smoothing parameters $\alpha ,\beta^{*}$, and γ are constrained, to ensure that the smoothing equations can be interpreted as weighted averages. Fitted values are calculated by setting h=1 with t=0,1,…,n−1. *H*-step ahead forecasts, for h=1,2,…, can then be obtained using the last estimated values of the level, trend, and seasonality (t=n). Details about all the other methods may be found in Hyndman and Athanasopoulos [18]. To be able to produce forecast intervals and use a model selection criteria, Hyndman et al. [19] (amongst others) developed a statistical framework, where an innovation state space model can be written for each of the exponential smoothing methods. Each state space model comprises a measurement equation, which describes the observed data, and state equations which describe how the unobserved components (level, trend, and seasonality) change with time. For each exponential smoothing method, two possible state space models are considered, one with additive errors and one with multiplicative errors, giving a total of 18 models. To distinguish state space models with additive and multiplicative errors, an extra letter E was added: The triplet $\left(E,T,S\right)$ identifies the type of error, trend, and seasonality. The general state space model is

(2a)yt=w(xt−1)+r(xt−1)εt(2b)xt=f(xt−1)+g(xt−1)εt,

where $y_{t}$ denotes the observation at time *t*, $x_{t}$ is the state vector, $\left\{\varepsilon_{t}\right\}$ is a white noise process with variance $\sigma^{2}$ referred to as the innovation (new and unpredictable), w(.) is the measurement function, r(.) is the error term function, f(.) is the transition function, and g(.) is the persistence function. Equation (2a) is the measurement equation and Equation (2b) gives the state equations. The measurement equation shows the relationship between the observations and the unobserved states. The transition equation shows the evolution of the state through time. The equations of the ETS$\left(A,A,A\right)$ model (underlying additive Holt-Winters’ method with additive errors) are [18]

(3a)yt=lt−1+bt−1+st−m+εt(3b)lt=lt−1+bt−1+αεt(3c)bt=bt−1+βεt(3d)st=st−m+γεt,

and the equations of the ETS$\left(M,A,A\right)$ model (underling additive Holt-Winters’ method with multiplicative errors) are [19]

(4a)yt=lt−1+bt−1+st−m1+εt(4b)lt=lt−1+bt−1+αlt−1+bt−1+st−mεt(4c)bt=bt−1+βlt−1+bt−1+st−mεt(4d)st=st−m+γlt−1+bt−1+st−mεt.

#### 2.1.1. Estimation of State Space Models

Maximum likelihood estimates of the parameters and initial states of the state space model (2) can be obtained by minimizing its likelihood. The probability density function for $y={\left(y_{1},\ldots ,y_{n}\right)}^{\prime }$ is given by [19]

(5) $$p\left(y\;|\;\theta ,x_{0},\sigma^{2}\right)=\prod_{t=1}^{n}p\left(y_{t}\;|\;x_{t-1}\right)=\prod_{t=1}^{n}p\left(\varepsilon_{t}\right)/\left|r\left(x_{t-1}\right)\right|,$$

where θ is the parameters vector, $x_{0}$ is the initial states vector, and $\sigma^{2}$ is the innovation variance. By assuming that the distribution of $\left\{\varepsilon_{t}\right\}$ is Gaussian, this likelihood has the form

(6) $$L\left(\theta ,x_{0},\sigma^{2}\;|\;y\right)={\left(2\pi \sigma^{2}\right)}^{-n/2}{\left|\prod_{t=1}^{n}r\left(x_{t-1}\right)\right|}^{-1}\exp\left(-\frac{1}{2}\sum_{t=1}^{n}\varepsilon_{t}^{2}/\sigma^{2}\right),$$

and its logarithm is

(7) $$\log L=-\frac{n}{2}\log\left(2\pi \sigma^{2}\right)-\sum_{t=1}^{n}\log\left|r(x_{t-1})\right|-\frac{1}{2}\sum_{t=1}^{n}\varepsilon_{t}^{2}/\sigma^{2}.$$

The maximum likelihood estimate of $\sigma^{2}$ can be obtained by taking the partial derivative of (7) with respect to $\sigma^{2}$ and setting it to zero:

(8) $${\hat{\sigma }}^{2}=n^{-1}\sum_{t=1}^{n}\varepsilon_{t}^{2}.$$

This estimate can be used to eliminate $\sigma^{2}$ from the likelihood (6), which becomes

(9) $$L\left(\theta ,x_{0}\;|\;y\right)={\left(2\;\pi \;e\;{\hat{\sigma }}^{2}\right)}^{-n/2}{\left|\prod_{t=1}^{n}r\left(x_{t-1}\right)\right|}^{-1}.$$

Hence, twice the negative logarithm of this likelihood is

(10) $$-2\log L\left(\theta ,x_{0}\;|\;y\right)=c_{n}+n\;\log\left(\sum_{t=1}^{n}\epsilon_{t}^{2}\right)+2\sum_{t=1}^{n}\log\left|r\left(x_{t-1}\right)\right|,$$

where $c_{n}=n\log\left(2\;\pi \;e\right)-n\log\left(n\right)$. Thus, maximum likelihood estimates for the parameters θ and the initial states $x_{0}$ can be obtained by minimizing

(11) $$L^{*}\left(\theta ,x_{0}\right)=n\;\log\left(\sum_{t=1}^{n}\epsilon_{t}^{2}\right)+2\sum_{t=1}^{n}\log\left|r\left(x_{t-1}\right)\right|.$$

The innovations can be computed recursively, using the relationships

(12) $$\begin{array}{cc} \;\varepsilon_{t} & =\left[y_{t}-w\left(x_{t-1}\right)\right]/r\left(x_{t-1}\right) \end{array}$$

(13) $$\begin{array}{cc} x_{t} & =f\left(x_{t-1}\right)+g\left(x_{t-1}\right)\varepsilon_{t}. \end{array}$$

#### 2.1.2. Information Criteria for Model Selection

Forecast accuracy measures can be used to select a model for a given time-series, as long as the errors are computed from a test set and not from the training set used to estimate the model. However, the errors usually available are not enough to draw reliable conclusions. One possible solution is to use an information criterion (IC), based on the likelihood $L(\theta ,x_{0}\;|\;y)$, that would include a regularization term to compensate for potential overfitting. The Akaike Information Criteria (AIC) for state space models is defined as [18]

(14) $$\mathrm{AIC}=-2\log L(\theta ,x_{0}\;|\;y)+2k,$$

where $L(\theta ,x_{0}\;|\;y)$ is the likelihood and *k* is the number of parameters and initial states of the estimated model. Akaike based his model selection criteria on the Kullback-Liebler (K-L) discrimination information, also known as negative entropy, defined by

(15) $$I\left(f,g\right)=\int f\left(x\right)\log\left(\frac{f(x)}{g(x|\theta )}\right)dx,$$

which measures the information lost when the model *g* is used to approximate the real model f. He found that he could estimate the expectation of K-L information by the maximized log-likelihood corrected for bias. This bias can be approximated by the number of estimated parameters in the approximating model. Thus, the model selection procedure is to choose the model amongst the candidates having the minimum value of the AIC. The Bayesian Information Criteria (BIC) is defined as [20]

(16) BIC=AIC+k[log(n)−2].

The BIC is order-consistent, but is not asymptotically efficient like the AIC. The AIC corrected for small-sample bias, denoted by ${\mathrm{AIC}}_{c}$, is defined as [19]

(17) $${\mathrm{AIC}}_{c}=\mathrm{AIC}+\frac{k(k+1)}{n-k-1}.$$

Appropriate models can be selected by minimizing the AIC, the BIC, or the ${\mathrm{AIC}}_{c}$.

### 2.2. ARIMA Models

ARIMA models are generally accepted as one of the most versatile classes of models for forecasting time-series [21,22]. Many different types of stochastic seasonal and non-seasonal time-series can be represented by them. These include pure autoregressive (AR), pure moving average (MA), and mixed AR and MA processes, all requiring stationary data so that they can be applied. Although many time-series are non-stationary, they can be transformed to stationary time-series by taking proper degrees of differencing (regular and/or seasonal). The multiplicative seasonal ARIMA model, denoted as ARIMA$\left(p,d,q\right)\times {\left(P,D,Q\right)}_{m}$, has the following form [23]:

(18) $$\phi_{p}\left(B\right)\Phi_{P}\left(B^{m}\right){\left(1-B\right)}^{d}{\left(1-B^{m}\right)}^{D}y_{t}=c+\theta_{q}\left(B\right)\Theta_{Q}\left(B^{m}\right)\varepsilon_{t},$$

where

$$\begin{array}{cccc} \phi_{p}\left(B\right) & =1-\phi_{1}B-\cdots -\phi_{p}B^{p} & \;\Phi_{P}\left(B^{m}\right) & =1-\Phi_{1}B^{m}-\cdots -\Phi_{P}B^{Pm},\\ \theta_{q}\left(B\right) & =1+\theta_{1}B+\cdots +\theta_{q}B^{q} & \;\Theta_{Q}\left(B^{m}\right) & =1+\Theta_{1}B^{m}+\cdots +\Theta_{Q}B^{Qm}, \end{array}$$

*m* is the period of seasonality, *D* is the degree of seasonal differencing, *d* is the degree of ordinary differencing, *B* is the backward shift operator, $\phi_{p}\left(B\right)$ and $\theta_{q}\left(B\right)$ are the regular autoregressive and moving average polynomials of orders *p* and *q*, respectively, $\Phi_{P}\left(B^{m}\right)$ and $\Theta_{Q}\left(B^{m}\right)$ are the seasonal autoregressive and moving average polynomials of orders *P* and *Q*, respectively, $c=\mu \left(1-\phi_{1}-\cdots -\phi_{p}\right)\left(1-\Phi_{1}-\cdots -\Phi_{P}\right)$, where μ is the mean of ${\left(1-B\right)}^{d}{\left(1-B^{m}\right)}^{D}y_{t}$, and $\varepsilon_{t}$ is a zero-mean Gaussian white noise process with variance $\sigma^{2}$. To ensure causality and invertibility, the roots of the polynomials $\phi_{p}\left(B\right)$, $\Phi_{P}\left(B^{m}\right)$, $\theta_{q}\left(B\right)$, and $\Theta_{Q}\left(B^{m}\right)$ should lie outside the unit circle. One of the main tasks in ARIMA forecasting is selecting the values of p,q,P,Q,d, and *D*. Usually, the following steps are used [23]: Plot the series, identify outliers, and choose a proper variance-stabilizing transformation. For that purpose, a Box-Cox transformation may be applied [24]:

(19) $$y_{t}^{\prime }=\left\{\begin{array}{cc} \ln(y_{t}), & \lambda =0\\ (y_{t}^{\lambda }-1)/\lambda , & \lambda \neq 0 \end{array}\right.,$$

where the parameter λ is a real number, often between −1 and 2. Then, the sample ACF (Auto-Correlation Function) and sample PACF (Partial Auto-Correlation Function) can be computed to decide appropriate degrees of differencing (*d* and *D*). Alternatively, unit-root tests may be applied. The Canova–Hansen test [25] can be used to choose *D*. After *D* is selected, *d* can be chosen by applying successive KPSS (Kwiatkowski, Phillips, Schmidt & Shin) tests [26]. Finally, the sample ACF and sample PACF are matched with the theoretical patterns of known models, to identify the orders of p,q,P, and *Q*.

#### Information Criteria for Model Selection

As for state space models, the values of p,q,P, and *Q* may be selected by an information criterion, such as the Akaike Information Criteria [18]:

(20) $$\mathrm{AIC}=-2\log L\left(\theta ,\;\sigma^{2}|\;y\right)+2\left(p+q+P+Q+k+1\right),$$

where k=1 if c≠0 and 0 otherwise, and $\log L(\theta ,\;\sigma^{2}|\;y)$ is the log-likelihood of the model fitted to the properly transformed and differenced data, given by [27]

(21) $$\log L\left(\theta ,\;\sigma^{2}|\;y\right)=-\frac{n}{2}\log\left(2\pi \right)-\frac{n}{2}\log\left(\sigma^{2}\right)-\sum_{t=1}^{n}\frac{\varepsilon_{t}^{2}}{2\sigma^{2}},$$

where θ is the parameter vector of the model and $\sigma^{2}$ is the innovation variance (the last term in parentheses in (20) is the total number of parameters that have been estimated, including the innovation variance). Note that the AIC is defined by considering the same principles of maximum likelihood and negative entropy discussed in Section 2.1. The AIC corrected for small sample sizes, ${\mathrm{AIC}}_{c}$, is defined as

(22) $${\mathrm{AIC}}_{c}=\mathrm{AIC}+\frac{2(p+q+P+Q+k+1)(p+q+P+Q+k+2)}{n-p-q-P-Q-k-2}.$$

The Bayesian Information Criterion is defined as

(23) BIC=AIC+[log(n)−2](p+q+P+Q+k+1).

As for the state space models, appropriate ARIMA models may be obtained by minimizing either the AIC, ${\mathrm{AIC}}_{c}$, or BIC.

## 3. Hierarchical Forecasting

### 3.1. Hierarchical Time-Series

**Figure 1:**
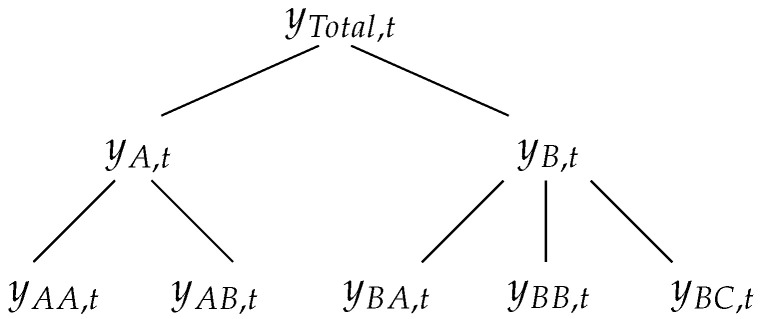
Example of a two-level hierarchical structure.

For the purpose of illustration, consider the example of the hierarchical structure shown in Figure 1. At the top of the hierarchy (level 0) is the most aggregated time-series, denoted by *Total*. The observation at time *t* of the *Total* series is denoted by $y_{Total,t}$. The *Total* series is disaggregated into series *A* and series *B*, at level 1. The *t*-th observation of series *A* is denoted as $y_{A,t}$ and the *t*-th observation of series *B* is denoted as $y_{B,t}$. The series *A* and *B* are disaggregated, respectively, into two and three series that are at the bottom level (level 2). For example, $y_{AA,t}$ denotes the *t*-th observation of series *AA*. In this case, the total number of series is n=8 and the number of series at the bottom level is m=5. For any time *t*, the observations at the bottom level will sum to the observations of the series above. Hence, in this case, we have

(24) $$y_{Total,t}=y_{AA,t}+y_{AB,t}+y_{BA,t}+y_{BB,t}+y_{BC,t},\;y_{A,t}=y_{AA,t}+y_{AB,t},\;y_{B,t}=y_{BA,t}+y_{BB,t}+y_{BC,t}.$$

These aggregation constraints can be easily represented using matrix notation

(25) $$y_{t}=Sb_{t},$$

where $y_{t}={\left(y_{Total,t},y_{A,t},y_{B,t},y_{AA,t},y_{AB,t},y_{BA,t},y_{BB,t},y_{BC,t}\right)}^{\prime }$ is an *n*-dimensional vector, $b_{t}={\left(y_{AA,t},y_{AB,t},y_{BA,t},y_{BB,t},y_{BC,t}\right)}^{\prime }$ is an *m*-dimensional vector, and S is the summing matrix of order n×m, given by

(26) $$S=\left[\begin{array}{ccccc} 1 & 1 & 1 & 1 & 1\\ 1 & 1 & 0 & 0 & 0\\ 0 & 0 & 1 & 1 & 1\\ & & I_{5} & & \end{array}\right].$$

Note that the first three rows of S correspond, respectively, to the three aggregation constraints in (24). The identity matrix $I_{5}$ below guarantees that each bottom level observation on the right-hand side of the equation is equal to itself on the left hand side. These concepts can be applied to an arbitrary set of *n* time-series that are subject to an aggregation structure, with *m* series at the bottom level [18]. The goal is to produce coherent forecasts for each series in the hierarchy; that is, forecasts that add up according to the aggregation constraints of the hierarchical structure.

### 3.2. Hierarchical Forecasting Methods

Let ${\hat{y}}_{t+h|t}$ be an *n*-dimensional vector containing the forecasts of the values of all series in the hierarchy at time t+h (with h=1,2,…), obtained using observations up to and including time *t*, and stacked in the same order as $y_{t}$. These are usually called base forecasts. They are calculated independently for each time-series, not taking into account any relationship that might exist between them due to the aggregation constraints. Any forecasting method, such as ETS or ARIMA, can be used to generate these forecasts. The issue is that it is very unlikely that these will be coherent forecasts, hence some reconciliation method should be further applied. All existing reconciliation methods can be expressed as

(27) $${\tilde{y}}_{t+h|t}=SP{\hat{y}}_{t+h|t},$$

where ${\tilde{y}}_{t+h|t}$ is an *n*-dimensional vector of reconciled forecasts, which are now coherent, and P is a matrix of dimension m×n, which maps the base forecasts ${\hat{y}}_{t+h|t}$ into reconciled bottom level forecasts, which are then aggregated by the summing matrix S. If the bottom-up (BU) approach is used, then $P=[0_{m\times (n-m)}\;|\;I_{m}]$, where $0_{m\times (n-m)}$ is the null matrix of order m×(n−m) and $I_{m}$ is the identity matrix of order *m* [4,5,6,9,10,28,29]. For the hierarchy shown in Figure 1, P is given by

(28) $$P=\left[\begin{array}{cccccccc} 0 & 0 & 0 & 1 & 0 & 0 & 0 & 0\\ 0 & 0 & 0 & 0 & 1 & 0 & 0 & 0\\ 0 & 0 & 0 & 0 & 0 & 1 & 0 & 0\\ 0 & 0 & 0 & 0 & 0 & 0 & 1 & 0\\ 0 & 0 & 0 & 0 & 0 & 0 & 0 & 1 \end{array}\right].$$

This approach is computationally very efficient, since it only requires summing the bottom level base forecasts. It also has the advantage of forecasting the series at the most disaggregated level and, although it is more difficult to model, no information about the dynamics of the series is lost due to aggregation. However, it usually provides very poor forecasts for the upper levels in the hierarchy [13]. If a top-down (TD) approach is used, then $P=[p\;|\;0_{m\times (n-1)}]$, where $p={\left[p_{1},\ldots ,p_{m}\right]}^{\prime }$ is an *m*-dimensional vector containing the disaggregation proportions, which indicate how the top level base forecast at time t+h is to be distributed to obtain forecasts for the bottom level series, which are then summed by S [8,17,30,31,32,33]. For the hierarchy shown in Figure 1, P is given by

(29) $$P=\left[\begin{array}{cccccccc} p_{1} & 0 & 0 & 0 & 0 & 0 & 0 & 0\\ p_{2} & 0 & 0 & 0 & 0 & 0 & 0 & 0\\ p_{3} & 0 & 0 & 0 & 0 & 0 & 0 & 0\\ p_{4} & 0 & 0 & 0 & 0 & 0 & 0 & 0\\ p_{5} & 0 & 0 & 0 & 0 & 0 & 0 & 0 \end{array}\right].$$

The most common top-down methods performed quite well in Gross and Sohl [8]. In method “a” of Gross and Sohl [8] (referred to in the results that follow as TD${}_{\mathrm{GSa}}$), each proportion $p_{i}$ is the average of the historical proportions of bottom level series $y_{i,j}$, relative to top level series $y_{T,j}$, over the time period j=1,…,t:

(30) $$p_{i}=\frac{1}{t}\sum_{j=1}^{t}\frac{y_{i,j}}{y_{T,j}},\;i=1,\ldots ,m.$$

In method “f” (referred to in the results that follow as TD${}_{\mathrm{GSf}}$), each proportion $p_{i}$ is the average value of the historical data of bottom level series $y_{i,j}$, relative to the average value of the historical data of top level series $y_{T,j}$, over the time period j=1,…,t:

(31) $$p_{i}=\sum_{j=1}^{t}\frac{y_{i,j}}{t}/\sum_{j=1}^{t}\frac{y_{T,j}}{t},\;i=1,\ldots ,m.$$

These two methods are very simple to implement, since they only require forecasts for the most aggregated series in the hierarchy. They seem to provide reliable forecasts for the aggregate levels. However, they are not able to capture the individual dynamics of the series that is lost due to aggregation. Moreover, since they are based on historical proportions, they tend to produce less accurate forecasts than the bottom-up approach at lower levels of the hierarchy, as they do not take into account how these proportions may change over time. To address this issue, Athanasopoulos et al. [9] proposed to obtain proportions based on forecasts rather than historical data:

(32) $$p_{i}=\prod_{l=0}^{k-1}\frac{{\hat{y}}_{i,t+h|t}^{\left(l\right)}}{{\hat{S}}_{i,t+h|t}^{\left(l+1\right)}},\;i=1,\ldots ,m,$$

where *k* is the level of the hierarchy, ${\hat{y}}_{i,t+h|t}^{\left(l\right)}$ is the base forecast at the time t+h of the series that corresponds to the node which is *l* levels above *i*, and ${\hat{S}}_{i,t+h|t}^{\left(l+1\right)}$ is the sum of the base forecasts at the time t+h of the series that corresponds to the nodes that are below the node that is *l* levels above node *i* and are directly connected to it. In the results that follow, this top-down method is referred as TD${}_{\mathrm{fp}}$. In the methods discussed so far, no real reconciliation has been performed, because these have been based on base forecasts from a single level of the hierarchy. However, processes that reconcile the base forecasts from the whole hierarchy structure in order to produce coherent forecasts can also be considered. Hyndman et al. [13] proposed an approach based on the regression model

(33) $${\hat{y}}_{t+h|t}=S\beta_{t+h|t}+\varepsilon_{h},$$

where $\beta_{t+h|t}$ is the unknown conditional mean of the most disaggregated series and $\varepsilon_{h}$ is the coherency error assumed with mean zero and covariance matrix $\Sigma_{h}$. If $\Sigma_{h}$ was known, the generalised least squares (GLS) estimator of $\beta_{t+h|t}$ would lead to the following reconciled forecasts

(34) $${\tilde{y}}_{t+h|t}=S{\hat{\beta }}_{t+h|t}=S{\left(S^{\prime }\Sigma_{h}^{-1}S\right)}^{-1}S^{\prime }\Sigma_{h}^{-1}{\hat{y}}_{t+h|t}=SP{\hat{y}}_{t+h|t},$$

where $P={\left(S^{\prime }\Sigma_{h}^{-1}S\right)}^{-1}S^{\prime }\Sigma_{h}^{-1}$. Hyndman et al. [13] also showed that, if the base forecasts ${\hat{y}}_{t+h|t}$ are unbiased, then the reconciled forecasts ${\tilde{y}}_{t+h|t}$ will be unbiased, provided that SPS=S. This condition is true for this reconciliation approach and also for the bottom-up, but not for top-down methods. So, the top-down approaches will never give unbiased reconciled forecasts, even if the base forecasts are unbiased. Recently, Wickramasuriya et al. [15] showed that, in general, $\Sigma_{h}$ is not identifiable. They showed that the covariance matrix of the *h*-step ahead reconciled forecast errors is given by

(35) $$\mathrm{Var}\left(y_{t+h}-{\tilde{y}}_{t+h|t}\right)=SPW_{h}P^{\prime }S^{\prime },$$

for any P such that SPS=S, where $W_{h}=\mathrm{Var}\left(y_{t+h}-{\hat{y}}_{t+h|t}\right)=E\left({\hat{e}}_{t+h|t}{\hat{e}}_{t+h|t}^{\prime }\right)$ is the covariance matrix of the corresponding *h*-step ahead base forecast errors. The goal is to find the matrix P that minimises the error variances of the reconciled forecasts, which are on the diagonal of the covariance matrix $\mathrm{Var}(y_{t+h}-{\tilde{y}}_{t+h|t})$. Wickramasuriya et al. [15] showed that the optimal reconciliation matrix P that minimises the trace of $SPW_{h}P^{\prime }S^{\prime }$, such that SPS=S, is

(36) $$P={\left(S^{\prime }W_{h}^{-1}S\right)}^{-1}S^{\prime }W_{h}^{-1}.$$

Therefore, the optimal reconciled forecasts are given by

(37) $${\tilde{y}}_{t+h|t}=S{\left(S^{\prime }W_{h}^{-1}S\right)}^{-1}S^{\prime }W_{h}^{-1}{\hat{y}}_{t+h|t},$$

which is referred to as the MinT (Minimum Trace) estimator. Note that the MinT and GLS estimators only differ in the covariance matrix. We still need to estimate $W_{h}$, which is a matrix of order *n* that can be quite large. The following simplifying approximations were considered by Wickramasuriya et al. [15]:

(1) $W_{h}=k_{h}I_{n}$ for all *h* with $k_{h}>0$. In this case, the MinT estimator corresponds to the ordinary least squares (OLS) estimator of $\beta_{t+h|t}$. It is the most simplifying approximation considered, being P-independent of the data (it only depends on S), which means that this method does not account for differences in scale between the levels of the hierarchy (captured by the error variances of the base forecasts), or the relationships between the series (captured by the error covariances of the base forecasts). This is optimal only when the base forecast errors are uncorrelated and equivariant, which are unrealistic assumptions for an hierarchical time-series. In the results that follow, this method is referred to as OLS.

(2) $W_{h}=k_{h}\mathrm{diag}\left({\hat{W}}_{1}\right)$ for all *h* with $k_{h}>0$, where ${\hat{W}}_{1}$ is the sample covariance estimator of the in-sample 1-step ahead base forecast errors. Then, $W_{h}$ is a diagonal matrix with the diagonal entries of ${\hat{W}}_{1}$, which are the variances of the in-sample 1-step ahead base forecast errors, stacked in the same order as $y_{t}$. This approximation scales the base forecasts, using the variance of the residuals. In the results that follow, this specification is referred to as MinT-VarScale.

(3) $W_{h}=k_{h}\Lambda$ for all *h* with $k_{h}>0$, and Λ=diag(S1) where 1 is a unit vector of dimension *n*. This method was proposed by Athanasopoulos et al. [34] for temporal hierarchies, and assumes that the bottom level base forecasts errors are uncorrelated between nodes and have variance $k_{h}$. Hence, the diagonal entries in Λ are the number of forecast error variances contributing to each node, stacked in the same order as $y_{t}$. This estimator only depends on the aggregation constraints, being independent of the data. Therefore, it is usually referred to as structural scaling, and we label it as MinT-StructScale. Notice that this specification only assumes equivariant base forecast errors at the bottom level, which is an advantage over OLS. It is particularly useful when the residuals are not available, which is the case when the base forecasts are generated by judgmental forecasting.

(4) $W_{h}=k_{h}{\hat{W}}_{1,D}^{*}$ for all *h* with $k_{h}>0$, where ${\hat{W}}_{1,D}^{*}=\lambda {\hat{W}}_{1,D}+\left(1-\lambda \right){\hat{W}}_{1}$ is a shrinkage estimator that shrinks the off-diagonal elements of ${\hat{W}}_{1}$ towards zero (while the diagonal elements remain unchanged), ${\hat{W}}_{1,D}$ is a diagonal matrix with the diagonal entries of ${\hat{W}}_{1}$, and λ is the shrinkage intensity parameter. By parameterizing the shrinkage in terms of variances and correlations, rather than variances and covariances, and assuming that the variances are constant, Schäfer and Strimmer [35] proposed the following shrinkage intensity parameter

(38) $$\hat{\lambda }=\frac{\sum_{i\neq j}\hat{\mathrm{Var}({\hat{r}}_{ij})}}{\sum_{i\neq j}{\hat{r}}_{ij}^{2}},$$

where ${\hat{r}}_{ij}$ is the $ij^{\mathrm{th}}$ element of ${\hat{R}}_{1}$, the sample correlation matrix of the in-sample 1-step ahead base forecast errors. In contrast to variance and structure scaling estimators, which are diagonal covariance estimators accommodating only differences in scale between the levels of the hierarchy, this shrinkage estimator, which is a full covariance estimator, also accounts for the relationships between the series, while the shrinkage parameter regulates the complexity of the matrix $W_{h}$. In the results that follow, this method is referred to as MinT-Shrink. In all estimators, $k_{h}$ is a proportionality constant that needs to be estimated only to obtain prediction intervals.

## 4. Empirical Study

### 4.1. Case Study Data

The Jerónimo Martins Group is an international company, based in Portugal, with 225 years of accumulated experience in the retail sector. Food distribution is its main business and represents more than 95% of their consolidated sales. In Portugal, it leads the supermarket segment through a supply chain called Pingo Doce. This empirical study was performed using a real database of product sales from one of the largest stores of Pingo Doce. The data were aggregated on a weekly basis and span the period between 3 January 2012 and 27 April 2015, comprising a total of 173 weeks. Only the products that have at least one sale every week were considered, since these are the most challenging for inventory planning. The hierarchical structure of products adopted by the retailer, from the top level to the bottom level, is: Store > Area > Division > Family > Category > Sub-category > SKU. The total number of time-series considered is 1751 (aggregated and disaggregated) and their split in the six levels of the hierarchy is summarised in Table 1. The most aggregated level, referred to as the top level, comprises the total sales at the store level. Level 1 comprises these sales disaggregated by the six main areas: Grocery, specialized perishables, non-specialized perishables, beverages, detergents and cleaning, and personal care. These are further disaggregated, at level 2, into 21 divisions; at level 3, into 73 families; at level 4, into 203 categories; at level 5, into 459 subcategories; and, at the bottom level, into 988 SKUs (Stock Keeping Units).

**Figure 2:**
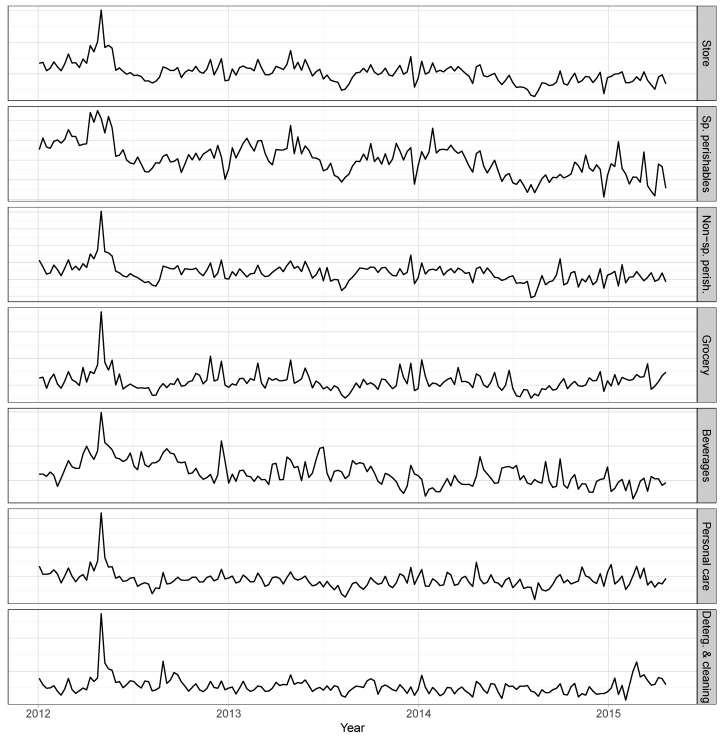
Total sales (top level, or store) and sales aggregated by area (level 1).

Figure 2 plots the sales at the top level and at level 1 of the hierarchy, aggregating these by the store and by each of the 6 main areas. The scale on the *y* axis was removed due to confidentiality reasons. The strong peak in sales in 2012, observed in all series, is relative to a promotional event carried out at a national level by Pingo Doce on 1 May (Labour day), after which the company shifted from an Every Day Low Price strategy to a continuous promotional cycle.

All the series show local upward and downward trends, although less prominent in the detergents/cleaning and personal care time-series. The store time-series shows a similar behaviour to the perishables time-series, as the later represent the major proportion of the total sales. These aggregate series do not show any seasonal variation.

**Figure 3:**
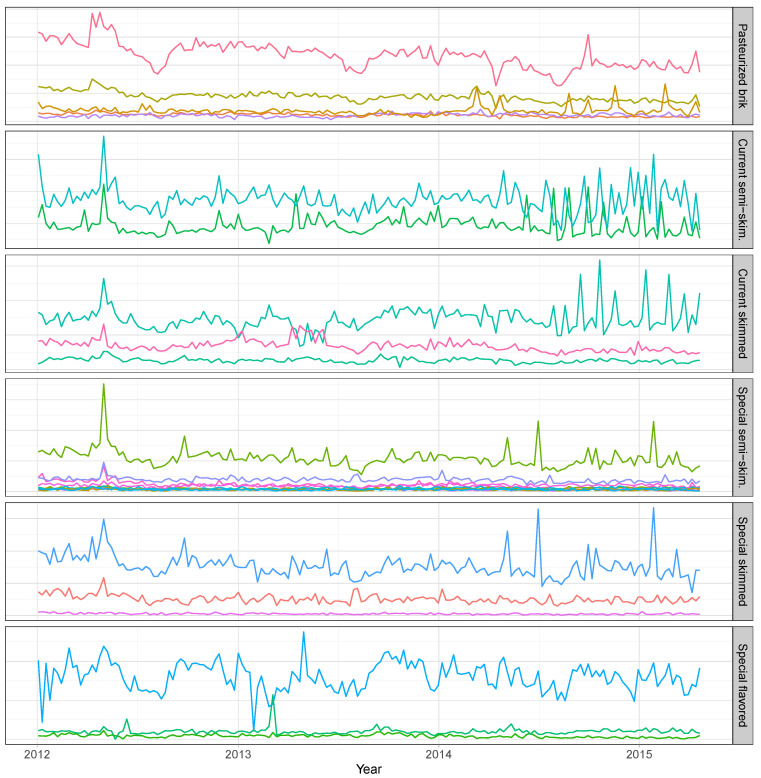
Sales of the SKUs within each sub-category of the milk division.

For a better understanding of the hierarchical structure of the data, we show, in Table 2, the complete hierarchy for the milk division (level 2). The total sales of the milk division are disaggregated, at level 3, into 2 families: Raw and UHT. The raw family is disaggregated into the Pasteurized category at level 4, which is further disaggregated into the Brik sub-category at level 5, which comprises 5 SKUs. The UHT family is disaggregated into the Current and Special categories. The Current category is disaggregated into the Semi-skimmed and Skimmed sub-categories, which comprise 2 and 3 SKUs, respectively. The Special category is disaggregated into the Semi-skimmed, Skimmed, and Flavored sub-categories, which comprise 10, 10, and 3 SKUs, respectively. The plots in Figure 3 show the sales of the SKUs within each subcategory of the milk division. These help us to visualise the diverse individual dynamics within each sub-category and the relative importance of each SKU. As we move down the hierarchy, the signal-to-noise ratio of the series decreases. Therefore, the series at the bottom level shows a lot more random variation, compared to the higher levels.

### 4.2. Experimental Setup

Generating accurate forecasts for each of the 1751 time-series within the hierarchical structure is crucial for the planning operations of the store. We can always forecast the series at each level of the hierarchy independently (we refer to these as base forecasts), based on forecasting models fitted individually for each series. However, by ignoring the aggregation constraints, it is very unlikely that the resulting forecasts will be coherent. To ensure aligned decision-making across the various levels of management, it is essential that these forecasts are reconciled across all levels of the hierarchy.

We consider two alternative forecasting model families for generating the base forecasts; namely, ETS and ARIMA, as discussed in Section 2. The appropriate ETS model for each time-series is chosen from the 18 potential models by minimising ${\mathrm{AIC}}_{c}$, and the smoothing parameters and initial states are estimated by maximising the likelihood L [19], as implemented in the forecast package in the R software [36]. The ARIMA model is chosen following the algorithm proposed by Hyndman and Khandakar [37], also implemented in the forecast package. First, the number of seasonal and ordinary differences *D* and *d* required for stationarity are selected, and then the orders of p,q,P, and *Q* are identified, based on ${\mathrm{AIC}}_{c}$. ETS and ARIMA models are the two most widely-used approaches to time-series forecasting. They are based on different perspectives to the problem and often, but not always, perform differently, although they share some mathematically equivalent models [21,22,38,39,40]. ARIMA can potentially capture higher-order time-series dynamics than ETS [34]. Therefore, we use both approaches to generate base forecasts, in order to evaluate how these can influence the performance of each reconciliation process. To make incoherent ETS and ARIMA forecasts coherent, we use the implementations of the hierarchical forecasting approaches, as discussed in Section 3.2, available in the hts package [41] for R.

**Figure 4:**
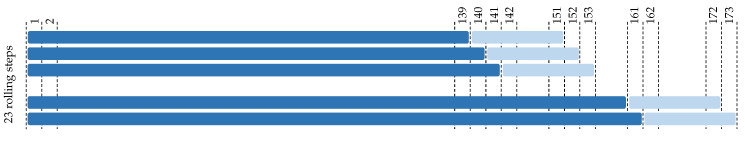
Cross-validation procedure, based on a rolling forecast origin with 1- to 12-week ahead forecasts.

We evaluate the forecasting accuracies of several competing methods using a rolling origin, as illustrated in Figure 4. By increasing the number of forecast errors available, we increase the confidence in our results.

We start with the training set containing the first 139 weeks and generate 1- to 12-week ahead base forecasts for each of the 1751 series using ETS and ARIMA. These base forecasts are then reconciled, using the alternative hierarchical methods. The training set is then expanded by one week, and the process is repeated until week 161. This gives a total of 23 forecast origins for each of the 1751 series. For each forecast origin, new ETS and ARIMA models based on the updated training data are specified, from which we generate new base forecasts which are again reconciled using the corresponding errors for both calculated. The performance of the hierarchical forecasting methods was evaluated by using the Average Relative Mean Squared Error (AvgRelMSE) [42]. As we are comparing forecast accuracy across time-series with different units, it is important to use a scale-independent error measure. For each time-series *i*, we calculate the Relative Mean Squared Error (RelMSE) [43]

(39) $${\mathrm{RelMSE}}_{i,h}=\frac{{\mathrm{MSE}}_{i,h}}{{\mathrm{MSE}}_{i,h}^{\mathrm{base}}},\;i=1,\ldots ,1751;\;\;h=1,2,4,8,12,$$

where ${\mathrm{MSE}}_{i,h}$ is the mean squared error of the forecast of interest averaged across all forecast origins and forecast horizons *h*, and ${\mathrm{MSE}}_{i,h}^{\mathrm{base}}$ is the mean squared error of the base forecast averaged across all forecast origins and forecast horizons *h*, which is used as a benchmark. If the hierarchical forecasting method reconciles with ARIMA (ETS) base forecasts, then the ARIMA (ETS) base forecasts are taken as the benchmark. For each forecast horizon *h*, we averaged (39) across the time-series of the hierarchy using the following geometric mean

(40) $${\mathrm{AvgRelMSE}}_{L,h}={\left(\prod_{i\in L}{\mathrm{RelMSE}}_{i,h}\right)}^{\frac{1}{\#L}},\;h=1,2,4,8,12.$$

where *L* is the level (i.e., Top level, Level 1, *…*, Level 5, Bottom level, All). The geometric mean should be used for averaging benchmark ratios, since it gives equal weight to reciprocal relative changes [44]. An advantage of AvgRelMSE is its interpretability. When it is smaller than 1, (1-AvgRelMSE)100% is the average percentage of improvement in MSE of the evaluated forecast over the benchmark.

### 4.3. Results

**Figure 5:**
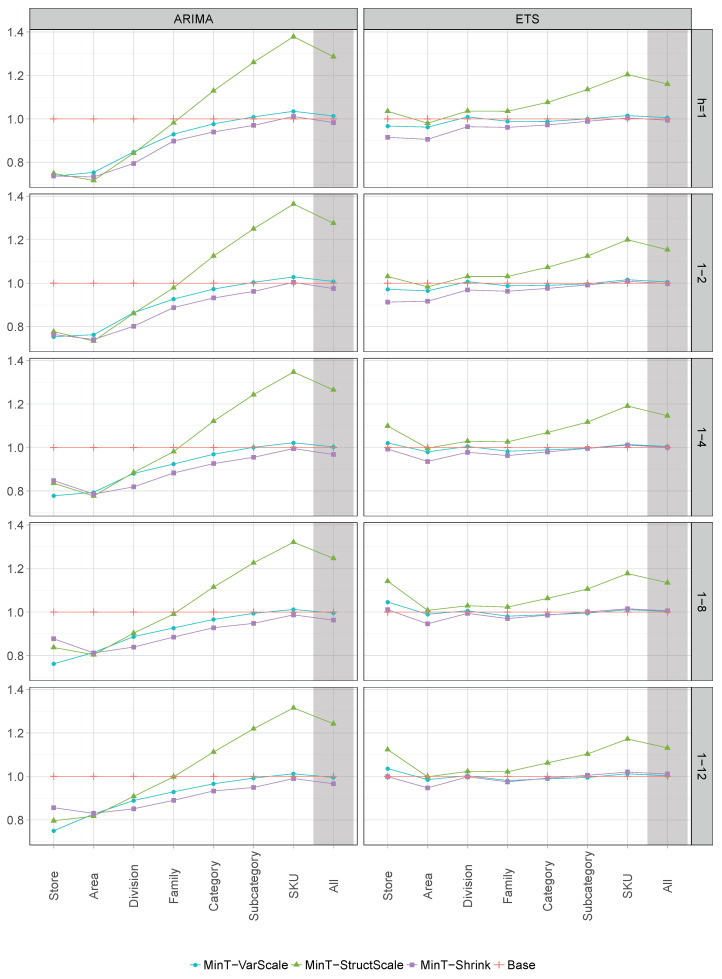
AvgRelMSE for the MinT-VarScale, MinT-StructScale, MinT-Shrink, and Base methods with ARIMA and ETS.

Table 3 presents the results of AvgRelMSE for the series of each hierarchical level, while Table 4 presents the results of AvgRelMSE for the complete hierarchy. BU refers to bottom-up method, TD${}_{\mathrm{GSa}}$ refers to top-down “a” method of Gross and Sohl [8], TD${}_{\mathrm{GSf}}$ refers to top-down “f” method of Gross and Sohl [8], TD${}_{\mathrm{fp}}$ refers to top-down with forecast proportions, OLS refers to Ordinary Least Squares, MinT-VarScale refers to Minimum Trace Variance Scaling estimator, MinT-StructScale refers to Minimum Trace Structural Scaling estimator, MinT-Shrink refers to Minimum Trace Shrinkage estimator and Base refers to base forecasts. The left side of these tables shows the results using ARIMA base forecasts, while the right side shows the results using ETS base forecasts. As the base forecasts were used to scale the errors, in the rows labelled Base the AvgRelMSE is equal to 1 across all columns. We provide forecast results for 1 week, 2 weeks, 4 weeks (about one month), 8 weeks (about two months), and 12 weeks (about three months). The column labelled Rank provides the mean rank of each method across all forecast horizons. A method with rank of 1 is interpreted as being the best on all the horizons, while that with a rank of 9 it is always the worst. To support the comparisons between the methods that are expected to perform better, Figure 5 visualises the results of AvgRelMSE for the MinT-VarScale, MinT-StructScale, MinT-Shrink, and Base methods, presented in the Table 3 and Table 4. The results for the complete hierarchy are highlighted with a light grey background.

It is immediately clear that the MinT-Shrink forecasts improved on the accuracy of the ARIMA base forecasts for all levels and for the complete hierarchy, across all forecast horizons. The only exception was the bottom level for the short-term horizons h=1 and 1−2(h=2), albeit with marginal differences. The gains in forecast accuracy were more substantial at the higher levels of aggregation. This was not the case for all other reconciliation methods, attesting to the difficulty of producing reconciled forecasts that were (at least) as accurate as the base forecasts. Furthermore, the MinT-Shrink method using ARIMA base forecasts returned the most accurate coherent forecasts for all levels, the only exceptions being the Store level, for which the MinT-VarScale returned the most accurate forecasts, and the Area level, where the MinT-StructScale performed best. The improvements on the accuracy of MinT-Shrink forecasts, across all forecast horizons, are more pronounced with the ARIMA base forecasts, compared to the ETS base forecasts (with the exception of horizon h=1 at the bottom level), although the former was almost always more accurate than the latter (see Table 5). This could have be due to the limitation of the ets() function in the forecast package, which restricts seasonality to have a maximum period of 24. Without this limitation, ARIMA can potentially capture seasonalities of a higher order than ETS.

Clearly, the least accurate method was the OLS, for both ETS and ARIMA forecasts and across all forecast horizons. OLS only improved forecast accuracy over the base forecasts at the top level. This was due to ignoring the differences in scale between the levels of the hierarchy and any relationships between the series. A major drawback of the TD${}_{\mathrm{GSa}}$ and TD${}_{\mathrm{GSf}}$ methods was that they only considered information from the top level. Interestingly, their forecasts only improved on the accuracy of the ARIMA base forecasts for the Area level, never improving over the ETS base forecasts (the forecasts at the top level are equal to the base forecasts). The TD${}_{\mathrm{fp}}$ proportions were based on forecasts from all disaggregated levels of the hierarchy, but it performed badly, never improving the forecast accuracy over the ARIMA base forecasts across all forecast horizons. This could be expected, since top-down approaches never give unbiased reconciled forecasts, even if the base forecasts are unbiased. BU provided poor forecasts for all aggregate levels in the hierarchy, showing average increases in the MSE relative to the base forecasts for all levels of aggregation and all forecast horizons (the forecasts at the bottom level are equal to the base forecasts). These losses in forecast accuracy were more substantial at higher levels of aggregation.

Like OLS, MinT-StructScale only depended on the structure of the aggregations and not on the actual data, resulting in poor forecasts, especially at the lower levels of aggregation; in our case, at the Category, Sub-category, and SKU levels, which comprised about 94% of the time-series of the complete hierarchy (see Figure 5). On the other hand, by accommodating the differences in scale between the levels of the hierarchy, MinT-VarScale performed well almost always, generally improving the forecast accuracy over the base forecasts. MinT-Shrink also accounted for the inter-relationships between the series in the hierarchy, always performing better than MinT-VarScale, across both ETS and ARIMA forecasts for all forecast horizons; the only exception being at the Store level (which comprised only one time-series).

To improve on the accuracy of the base forecasts, the reconciliation methods have to take advantage of the combination of informative signals from all levels of aggregation. It is clear that MinT-Shrink was able do this and, hence, improvements in forecast accuracy over the base forecasts were attained. For the complete hierarchy, the accuracy gains generally increased with the forecast horizon varying between 1.7% and 3.7%. It is also evident that the gains in forecast accuracy were more substantial at higher levels of aggregation, which means that information about the individual dynamics of the series which was lost due to aggregation, was brought back again from the lower levels of aggregation to the higher levels by the reconciliation process, substantially improving the forecast accuracy over the base forecasts.

These results are in accordance with those obtained by Kourentzes and Athanasopoulos [45], which compared MinT-Shrink and MinT-VarScale forecasts with base forecasts in the context of generating coherent cross-temporal forecasts for Australian tourism. Both MinT-Shrink and MinT-VarScale improved the forecast accuracy over the base ETS and ARIMA forecasts for the bottom level and the complete hierarchy. MinT-Shrink performed better than MinT-VarScale across both ETS and ARIMA forecasts.

**Figure 6:**
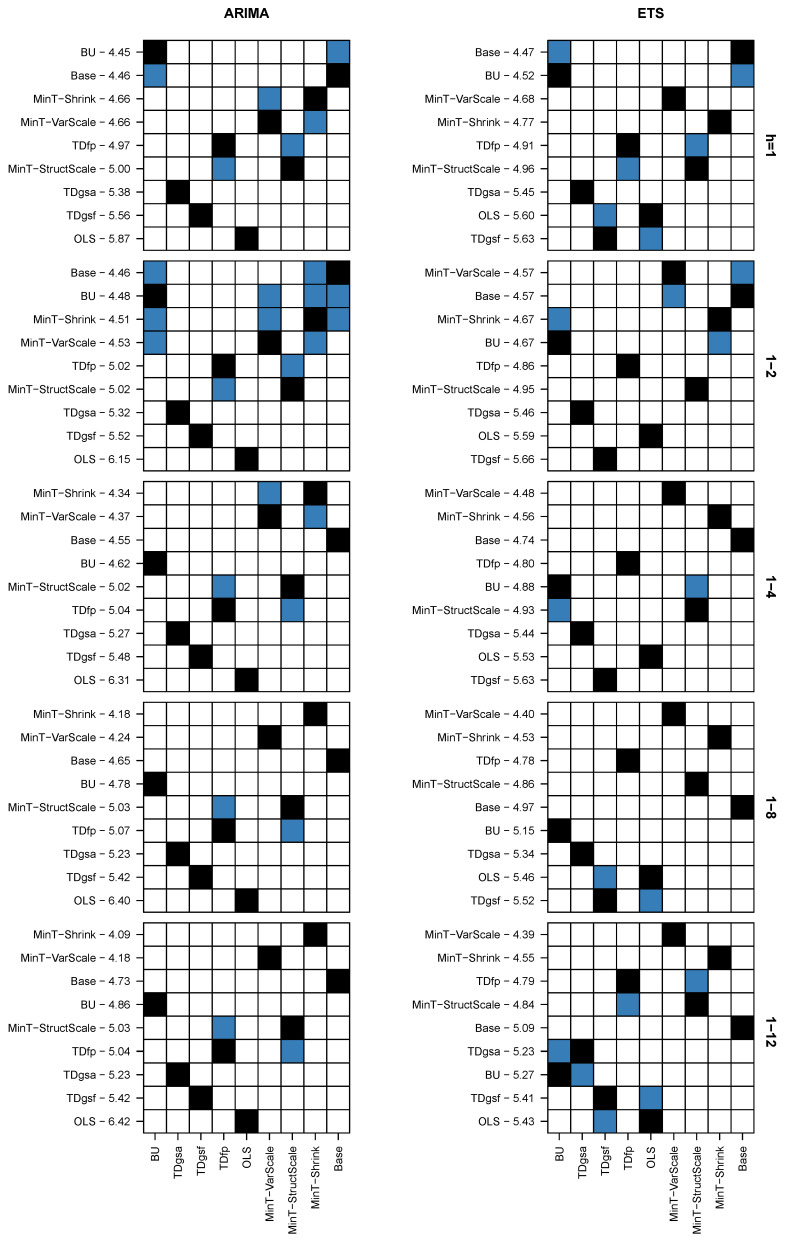
Nemenyi test results, at a 5% significance level, for the complete hierarchy.

In order to find out if the forecast error differences between the several competing methods were statistically significant or not, we conducted a Nemenyi test [46]. The results of this test are shown in Figure 6. The panels on the left side show the results for the complete hierarchy using ARIMA base forecasts, for each forecast horizon; while the panels on the right side show the respective results using ETS base forecasts. In the vertical axis, the methods are sorted by MSE mean rank. In the horizontal axis, they are ordered as in Table 3 and Table 4. In each row, the cell in black represents the method being tested and any blue cell indicates a method with no evidence of statistically significant differences, at a 5% level, while the white cells indicate methods without such evidence. We use the Nemenyi test implementation available in the tsutils [47] package for R.

Analysing the results for ARIMA presented in the panels on the left side, we observe that, for h=1, BU and Base are grouped together as the top-performing methods. They are immediately followed by MinT-Shrink and MinT-VarScale, which are found to be statistically indifferent. For the forecast horizon 1−2(h=2), BU, Base, MinT-Shrink, and MinT-VarScale are now grouped together as top-performing methods. For the forecast horizon 1−4(h=4), MinT-Shrink and MinT-VarScale belong to the top-performing group of forecasts and BU and Base perform significantly worse. For the long-term forecasts, MinT-Shrink performs significantly better than MinT-VarScale, BU, and Base. The TD${}_{\mathrm{fp}}$ and MinT-StructScale methods perform significantly worse than MinT-Shrink, MinT-VarScale, BU, and Base across all forecast horizons, and are found to be statistically indifferent, outperforming only TD${}_{\mathrm{GSa}}$, TD${}_{\mathrm{GSf}}$, and OLS.

Analysing the results for ETS presented in the panels on the right side, we observe that, for h=1, BU and Base are again grouped together as top-performing methods, followed by MinT-VarScale and MinT-Shrink. For the forecast horizon 1−2(h=2), MinT-VarScale and Base are grouped together as top-performing methods, being immediately followed by MinT-Shrink and BU; which are found to be statistically indifferent. For the other forecast horizons, MinT-VarScale performs better, being always followed by MinT-Shrink. Overall, for both ETS and ARIMA, the MinT approach outperforms the other competing methods, with the exception for the short horizon h=1.

## 5. Conclusions

Retailers need forecasts for a huge number of related time-series which can be organised into an hierarchical structure. Sales at the SKU level can be naturally aggregated into categories, families, areas, stores, and regions. To ensure aligned decision-making across the hierarchy, it is essential that forecasts at the most disaggregated level add up to forecasts at the aggregate levels above. It is not immediately clear if these aggregate forecasts should be generated independently or by using an hierarchical forecasting method that ensures coherent decision-making at the different levels but does not guarantee (at the least) the same accuracy. To give guidelines on this issue, our empirical study investigates the relative performance of independent and reconciled forecasting approaches.

We use weekly data of SKU sales from one big store of a Portuguese retailer, spanning the period between 3 January 2012 and 27 April 2015, and consider the hierarchical structure of products adopted by the company from the top level to the bottom level, comprising six levels overall. We generate the independent forecasts using two alternative forecasting model families; namely, ETS and ARIMA. These are compared to the most commonly-used hierarchical forecasting approaches. We evaluate the forecast accuracies of several competing methods, through the Average Relative Mean Squared Error, by using a cross-validation based on a rolling forecast origin.

It is clear that MinT-Shrink forecasts generally improve on the accuracy of the ARIMA base forecasts for all levels and for the complete hierarchy, across all forecast horizons. The accuracy gains generally increase with the horizon, varying between 1.7% and 3.7% for the complete hierarchy. That is not the case for all other reconciliation methods, attesting to the difficulty of producing reconciled forecasts that are at least as accurate as base forecasts. The improvements on the accuracy of MinT-Shrink forecasts, across all forecast horizons, are more pronounced with the ARIMA base forecasts, compared to the ETS base forecasts (with the exception to horizon h=1 at the bottom level); although, the former is almost always more accurate than the latter.

To improve on the accuracy of the base forecasts, the reconciliation methods have to take advantage of the combination of informative signals from all levels of aggregation. It is clear that MinT-Shrink is able do this and, hence, improvements in forecast accuracy over the base forecasts are attained. It is also evident that the gains in forecast accuracy are more substantial at higher levels of aggregation, which means that the information about the individual dynamics of the series lost when aggregating, is brought back again from the lower levels of aggregation to the higher levels by the reconciliation process, substantially improving the forecast accuracy over the base forecasts.

## Figures and Tables

**Table 1:** Number of series in each hierarchical level by area.

Area	Divisions	Families	Categories	Subcategories	SKUs
Specialized perishables	6	19	50	102	193
Non-specialized perishables	4	16	48	117	287
Grocery	3	14	51	144	309
Beverages	4	6	16	32	103
Personal care	2	9	19	37	59
Detergents & cleaning	2	9	19	27	37
Total	21	73	203	459	988

**Table 2:** Hierarchical structure of the milk division.

Area	Division	Families	Categories	Subcategories	SKUs
Non-specialized perishables	Milk	Raw	Pasteurized	Brik	5
		UHT	Current	Semi-skimmed	2
				Skimmed	3
			Special	Semi-skimmed	10
				Skimmed	3
				Flavored	3

**Table 3:** Average Relative Mean Squared Error (AvgRelMSE) for each level of the hierarchy obtained with ARIMA and ETS base forecasts.

	ARIMA							ETS					
Method	h=1	1−2	1−4	1−8	1−12	Rank		h=1	1−2	1−4	1−8	1−12	Rank
	Top-level: Store												
BU	2.074	2.179	2.489	2.569	2.237	9		1.748	1.721	1.869	1.990	1.914	9
TD${}_{\mathrm{GSa}}$	1	1	1	1	1	6.5		1	1	1	1	1	4.6
TD${}_{\mathrm{GSf}}$	1	1	1	1	1	6.5		1	1	1	1	1	4.6
TD${}_{\mathrm{fp}}$	1	1	1	1	1	6.5		1	1	1	1	1	4.6
OLS	0.949	0.951	0.959	0.950	0.947	4		0.985	0.990	0.998	1	0.999	2.5
MinT-VarScale	0.736	0.754	0.778	0.762	0.750	1		0.967	0.972	1.021	1.046	1.036	5
MinT-StructScale	0.749	0.777	0.836	0.837	0.796	2.4		1.035	1.031	1.099	1.142	1.123	8
MinT-Shrink	0.737	0.764	0.848	0.877	0.856	2.6		0.915	0.913	0.993	1.011	0.999	2.1
Base	1	1	1	1	1	6.5		1	1	1	1	1	4.6
	Level 1: Area												
BU	1.096	1.154	1.242	1.274	1.268	8.6		1.264	1.274	1.314	1.327	1.288	9
TD${}_{\mathrm{GSa}}$	0.895	0.899	0.922	0.950	0.972	5		1.077	1.074	1.069	1.092	1.083	7.6
TD${}_{\mathrm{GSf}}$	0.886	0.888	0.911	0.938	0.961	4		1.067	1.063	1.057	1.080	1.071	6.6
TD${}_{\mathrm{fp}}$	1.020	1.012	1.002	1.009	1.015	7		1.021	1.009	0.998	0.998	0.998	4.5
OLS	1.189	1.186	1.150	1.134	1.125	8.4		1.123	1.079	1.004	0.990	0.977	5.3
MinT-VarScale	0.754	0.763	0.794	0.814	0.827	2.8		0.962	0.965	0.980	0.990	0.985	2.3
MinT-StructScale	0.717	0.734	0.777	0.804	0.817	1		0.980	0.983	0.997	1.008	0.998	3.9
MinT-Shrink	0.733	0.741	0.786	0.812	0.830	2.2		0.906	0.917	0.936	0.946	0.947	1
Base	1	1	1	1	1	6		1	1	1	1	1	4.8
	Level 2: Division												
BU	1.082	1.131	1.175	1.212	1.192	7.6		1.278	1.278	1.277	1.256	1.227	8
TD${}_{\mathrm{GSa}}$	1.081	1.098	1.130	1.146	1.138	5.8		1.259	1.219	1.190	1.156	1.132	6
TD${}_{\mathrm{GSf}}$	1.089	1.104	1.136	1.151	1.142	7		1.269	1.226	1.197	1.162	1.137	7
TD${}_{\mathrm{fp}}$	1.091	1.091	1.082	1.068	1.056	5.6		1.026	1.020	1.002	1.004	1.006	3.6
OLS	1.966	1.953	1.994	2.029	2.027	9		1.523	1.495	1.461	1.471	1.457	9
MinT-VarScale	0.848	0.864	0.881	0.887	0.889	2.4		1.009	1.007	1.005	1.006	1.003	3.4
MinT-StructScale	0.842	0.861	0.885	0.903	0.908	2.6		1.036	1.031	1.029	1.029	1.023	5
MinT-Shrink	0.795	0.802	0.819	0.839	0.851	1		0.964	0.969	0.978	0.994	0.998	1
Base	1	1	1	1	1	4		1	1	1	1	1	2
	Level 3: Family												
BU	1.016	1.022	1.031	1.040	1.036	5		1.083	1.083	1.073	1.067	1.061	6.4
TD${}_{\mathrm{GSa}}$	1.194	1.182	1.174	1.155	1.130	7		1.217	1.176	1.132	1.079	1.043	6.8
TD${}_{\mathrm{GSf}}$	1.200	1.188	1.179	1.159	1.134	8		1.223	1.181	1.136	1.083	1.046	7.8
TD${}_{\mathrm{fp}}$	1.101	1.094	1.079	1.079	1.075	6		1.024	1.018	1.008	1.005	1.005	4
OLS	2.348	2.314	2.338	2.405	2.399	9		1.567	1.542	1.533	1.524	1.503	9
MinT-VarScale	0.930	0.927	0.924	0.927	0.929	2		0.989	0.988	0.983	0.981	0.981	2
MinT-StructScale	0.982	0.979	0.981	0.991	0.998	3		1.035	1.031	1.026	1.023	1.021	5
MinT-Shrink	0.898	0.888	0.883	0.885	0.890	1		0.961	0.963	0.963	0.970	0.975	1
Base	1	1	1	1	1	4		1	1	1	1	1	3
	Level 4: Category												
BU	1.014	1.015	1.019	1.029	1.029	4		1.027	1.028	1.027	1.028	1.027	4.2
TD${}_{\mathrm{GSa}}$	1.300	1.290	1.271	1.249	1.233	7		1.295	1.263	1.219	1.159	1.122	7
TD${}_{\mathrm{GSf}}$	1.306	1.296	1.276	1.253	1.237	8		1.302	1.269	1.224	1.163	1.125	8
TD${}_{\mathrm{fp}}$	1.129	1.121	1.108	1.107	1.103	5.1		1.033	1.031	1.028	1.027	1.030	4.8
OLS	2.463	2.418	2.403	2.398	2.375	9		1.636	1.618	1.602	1.563	1.537	9
MinT-VarScale	0.977	0.973	0.969	0.966	0.966	2		0.988	0.990	0.989	0.988	0.989	1.8
MinT-StructScale	1.129	1.125	1.121	1.115	1.112	5.9		1.076	1.073	1.069	1.063	1.062	6
MinT-Shrink	0.940	0.932	0.926	0.928	0.933	1		0.972	0.976	0.980	0.986	0.992	1.2
Base	1	1	1	1	1	3		1	1	1	1	1	3
	Level 5: Subcategory												
BU	1.008	1.009	1.012	1.015	1.014	3.8		1.011	1.009	1.009	1.009	1.009	4
TD${}_{\mathrm{GSa}}$	1.326	1.301	1.274	1.231	1.208	6.8		1.314	1.270	1.220	1.155	1.117	7
TD${}_{\mathrm{GSf}}$	1.335	1.309	1.282	1.238	1.215	7.8		1.323	1.278	1.228	1.161	1.123	8
TD${}_{\mathrm{fp}}$	1.155	1.143	1.131	1.122	1.115	5		1.052	1.046	1.044	1.039	1.039	5
OLS	2.478	2.426	2.408	2.378	2.353	9		1.677	1.651	1.626	1.582	1.558	9
MinT-VarScale	1.009	1.004	1.001	0.994	0.992	2.8		1.000	0.997	0.997	0.995	0.995	1.7
MinT-StructScale	1.260	1.250	1.243	1.225	1.219	6.4		1.135	1.125	1.117	1.106	1.103	6
MinT-Shrink	0.970	0.962	0.955	0.948	0.949	1		0.989	0.992	0.996	1.001	1.005	1.8
Base	1	1	1	1	1	2.4		1	1	1	1	1	2.5
	Bottom-level: SKU												
BU	1	1	1	1	1	2.1		1	1	1	1	1	1.5
TD${}_{\mathrm{GSa}}$	1.381	1.355	1.321	1.267	1.243	6.2		1.387	1.346	1.293	1.217	1.177	7
TD${}_{\mathrm{GSf}}$	1.393	1.366	1.331	1.276	1.251	7.4		1.398	1.357	1.303	1.225	1.184	8
TD${}_{\mathrm{fp}}$	1.182	1.166	1.148	1.129	1.126	5		1.080	1.079	1.075	1.068	1.069	5
OLS	2.077	2.038	2.009	1.972	1.959	9		1.506	1.496	1.479	1.448	1.433	9
MinT-VarScale	1.035	1.029	1.022	1.012	1.012	4		1.015	1.016	1.014	1.011	1.012	3.6
MinT-StructScale	1.378	1.364	1.347	1.320	1.315	7.4		1.204	1.200	1.191	1.177	1.172	6
MinT-Shrink	1.011	1.004	0.995	0.987	0.990	1.8		1.004	1.009	1.011	1.015	1.020	3.4
Base	1	1	1	1	1	2.1		1	1	1	1	1	1.5

**Table 4:** AvgRelMSE for the complete hierarchy obtained with ARIMA and ETS base forecasts.

	ARIMA							ETS					
Method	h=1	1−2	1−4	1−8	1−12	Rank		h=1	1−2	1−4	1−8	1−12	Rank
	All												
BU	1.006	1.008	1.010	1.013	1.012	3.7		1.013	1.013	1.013	1.012	1.012	4
TD${}_{\mathrm{GSa}}$	1.343	1.320	1.292	1.248	1.225	6.8		1.346	1.306	1.256	1.186	1.148	7
TD${}_{\mathrm{GSf}}$	1.353	1.329	1.301	1.255	1.232	7.8		1.356	1.315	1.264	1.193	1.154	8
TD${}_{\mathrm{fp}}$	1.163	1.150	1.134	1.121	1.117	5		1.064	1.061	1.057	1.052	1.052	5
OLS	2.223	2.182	2.159	2.132	2.116	9		1.565	1.549	1.530	1.496	1.477	9
MinT-VarScale	1.013	1.008	1.003	0.996	0.995	2.9		1.006	1.006	1.005	1.003	1.003	2.6
MinT-StructScale	1.286	1.276	1.265	1.246	1.242	6.4		1.160	1.154	1.146	1.135	1.131	6
MinT-Shrink	0.983	0.975	0.968	0.963	0.966	1		0.994	0.998	1.001	1.006	1.011	2
Base	1	1	1	1	1	2.4		1	1	1	1	1	1.4

**Table 5:** AvgRelMSE results of ARIMA base forecasts with ETS base forecasts used as benchmark.

	h=1	1−2	1−4	1−8	1−12
Top-level	0.592	0.572	0.549	0.563	0.617
Level 1	1.007	0.98	0.958	0.947	0.929
Level 2	1.075	1.01	0.962	0.914	0.913
Level 3	0.986	0.961	0.931	0.902	0.894
Level 4	0.985	0.967	0.95	0.921	0.905
Level 5	0.984	0.969	0.955	0.937	0.925
Bottom-level	1.007	0.998	0.987	0.972	0.961
All	0.998	0.985	0.971	0.953	0.941
